# Surgical debridement of low velocity ballistic traumatic arthrotomy did not reduce rates of early wound infection or septic arthritis

**DOI:** 10.1007/s00590-025-04648-z

**Published:** 2026-01-07

**Authors:** Vishal S. Patel, Max Yang, Andrew Duong, Soroush Shabani, Daniel Rusu, Joseph T. Patterson

**Affiliations:** Keck School of Medicine of University of Southern California, Los Angeles, USA

**Keywords:** Arthrotomy, Ballistic, Gunshot, Debridement, Septic arthritis, Surgical site infection

## Abstract

**Purpose:**

To compare the incidence of ballistic wound infection and septic arthritis after operative versus nonoperative management of low-velocity ballistic traumatic arthrotomy of a major or intermediate joint.

**Methods:**

A retrospective cohort of consecutive adults treated within 24 h of injury at one Level 1 Trauma Center of low-velocity ballistic traumatic arthrotomy of the shoulder, elbow, wrist, hip, knee, or ankle from 2019 to 2023 was identified from an orthopedic consult registry. Treatment was classified as antibiotic therapy with or without formal surgical debridement with joint irrigation. The primary outcomes of ballistic wound infection and septic arthritis were compared by Fisher’s exact test with Šidák correction.

**Results:**

Five hundred seventy-eight patients with ballistic extremity injuries were screened. Seventy-seven patients met inclusion criteria. Thirty-five patients (45.5%) received operative care and 42 patients (54.5%) received nonoperative care. No significant differences were observed between treatment groups by age, sex, race/ethnicity, body mass index, area deprivation index (ADI), insurance status, medical comorbidities, the joint involved, or antibiotic regimen. No statistically significant difference in the rates of ballistic wound infection (2.4% vs. 5.7%, p = 0.830) or septic arthritis (4.8% vs. 8.5%, p = 0.995) were found between antibiotic therapy alone versus formal surgical debridement with joint irrigation.

**Conclusion:**

Formal surgical debridement of ballistic traumatic arthrotomy of large and intermediate joints did not provide incremental benefit over antibiotic therapy alone.

**Level of evidence:**

Therapeutic level IV.

## Introduction

Low-velocity ballistic injuries from small firearms were the mechanism of injury for over 46,728 deaths across the United States (U.S) in 2023 [1]. The incidence of ballistic fractures has increased more than 500% from the year 2000 to 2019 across the U.S. and a similar increase has been seen in Europe [2, 3]. Ballistic injuries to the extremities often produce complex fractures, soft tissue damage, and joint injuries. A ballistic traumatic arthrotomy occurs when a projectile breaches the capsule of a joint and may carry an increased risk of septic arthritis due to contamination of the immune privileged articular space with skin flora and foreign material [4].

Septic arthritis may have severe consequences including permanent loss of function in 25–50% of cases and even mortality related to secondary systemic infection reported in up to 10% of patients [5, 6]. The gold standard of treatment for blunt and penetrating traumatic arthrotomy of large and intermediate sized joints is incisional debridement and irrigation of the joint to remove contaminants [7–9]. Absolute indications for surgical management of ballistic traumatic arthrotomies included open fractures with contaminated tissue requiring extensive debridement not feasible at the bed side, neurovascular injuries requiring exploration or repair, fractures requiring fixation, and large ballistic foreign body fragments or loose osteoarticular fragments retained within the joint [7, 10]. However, a strong association between low velocity ballistic traumatic arthrotomy and infection has not been established. Some authors have questioned the necessity of routine surgical debridement for ballistic traumatic arthrotomy, suggesting that nonoperative management with antibiotics alone may be sufficient in certain cases [7, 11–13].

The purpose of this study was to evaluate the benefit of formal surgical debridement as an adjunct to antibiotic therapy for preventing infection after low-velocity ballistic traumatic arthrotomy of large and intermediate joints. We hypothesized that surgical debridement would be associated with a lower incidence of ballistic wound infection (BWI) and septic arthritis (SA).

## Patients and methods

A retrospective cohort study was conducted at one Level 1 trauma center. Patients 18 years of age and older who were treated for low-velocity ballistic traumatic arthrotomy of the shoulder, elbow, wrist, hip, knee, or ankle between 2019 and 2023 were identified from an orthopaedic consult registry. Data were collected from electronic medical records. Traumatic arthrotomy was defined by a positive saline load test or visualization of capsular rupture, intraarticular fracture, metallic debris on either radiographs or computed tomography [14, 15]. Low-velocity ballistic mechanism was confirmed by cross-referencing the institutional trauma registry, which adheres to the National Trauma Data Standards Data definitions [16]. Patients with a history of previous surgery or infection of the injured joint were excluded.

Treatment was classified as antibiotic therapy alone with bedside irrigation versus antibiotic therapy with adjunctive surgical debridement and irrigation performed in the operating room. Patients routinely received irrigation of ballistic wounds at bedside with dressing application on evaluation during the study period. Antibiotic therapy agent, route, and duration were recommended by the treating orthopaedic surgeon at their discretion and initiated by the acute care surgery service, ostensibly based on patient and injury factors. The indication of ballistic injuries with small retained foreign bodies for surgical debridement varied within and between surgeons during the study period, permitting investigation of this practice variation.

The primary outcomes included BWI and SA. BWI was defined by warmth, erythema, pain, and/or cloudy drainage located at the ballistic wound(s) within 30 days of presentation, without direct involvement of the joint documented by arthrocentesis or subsequent arthrotomy for irrigation and drainage of septic arthritis with negative cultures, and independent of subsequent operative debridement [17]. SA was defined as an inflammation of the affected joint secondary to an infection that was confirmed with synovial fluid analysis revealing a synovial white blood cell count greater than 50,000 cells/mm^3^, a 90% neutrophil predominance, and/or identification of a bacterial organism in the aspirated synovial fluid or from operative cultures obtained during debridement, irrigation, and drainage of the joint for septic arthritis [18, 19].

Demographic characteristics, medical conditions, insurance type, affected joint, and antibiotic therapy were compared between groups using two-tailed t-tests for continuous variables and Fisher exact tests for categorical variables. The incidence of BWI and SA were compared between the treatment groups using Fisher’s exact test without adjustment. Šidák-Holm corrections of 0.6 were applied to *p*-values reported for outcome comparisons given multiple hypothesis testing of two ostensibly correlated outcomes. A significance level of *p* < 0.05 was used for all analyses. Analyses were performed with R version 4.5.1 (R Foundation, Viena, Austria).

## Results

### Participant characteristics

**Fig. 1:**
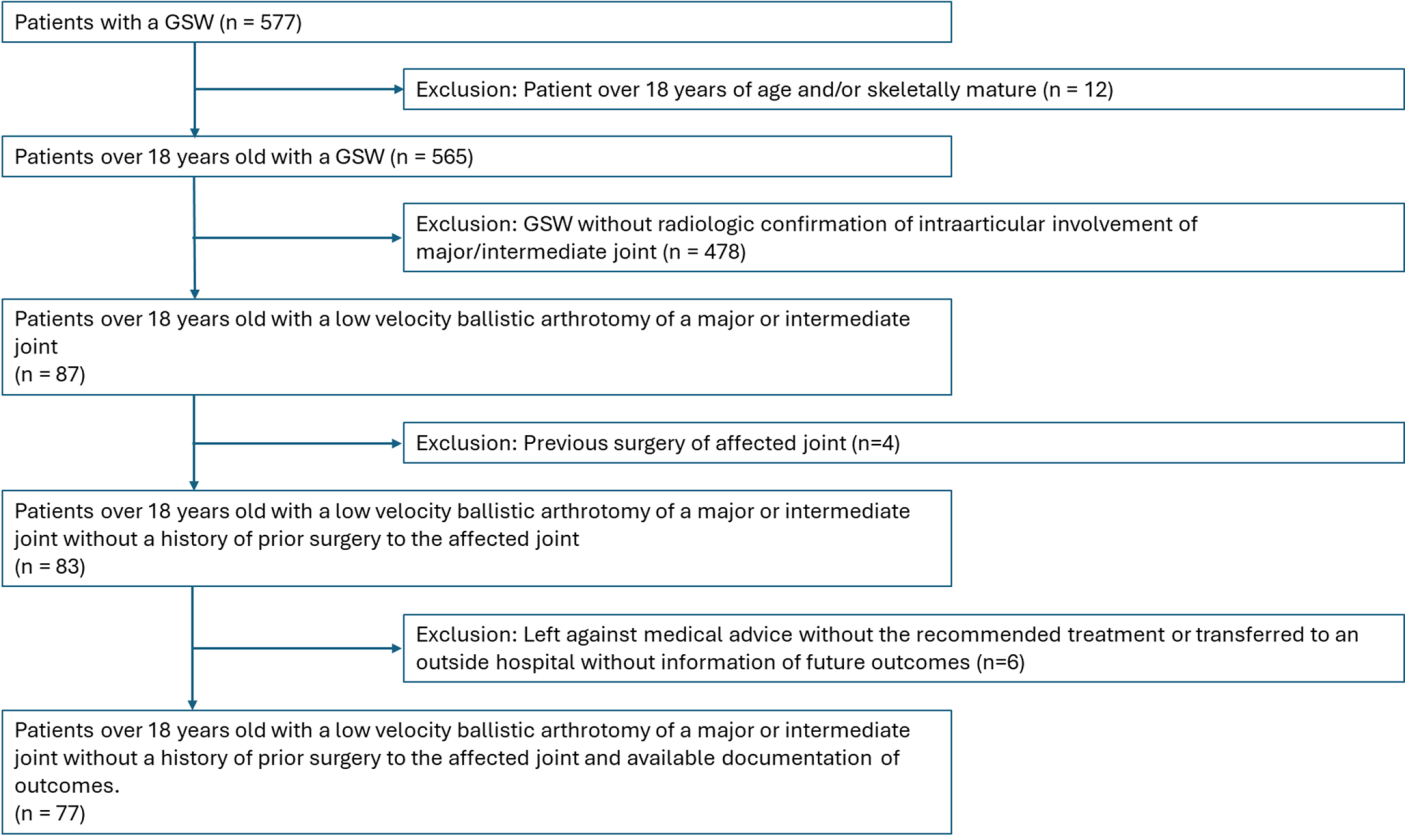
STROBE diagram of patient selection

Five hundred seventy-seven patients sustained a low-velocity ballistic wound during the observed period. Four hundred seventy-eight patients were excluded due to a lack of an affected major or intermediate joint. Twelve patients were excluded for being under eighteen years of age. Four patients were excluded due to a previous surgery of the affected joint. Eighty-three patients met the final inclusion criteria. Four patients were excluded from analysis because they left against medical advice without receiving the recommended treatment. Two patients were excluded because they were transferred to an outside hospital with no information about future outcomes. Of the seventy-seven included patients, 42 (54.5%) received antibiotic therapy alone with bedside irrigation and 35 (45.5%) received adjunctive surgical debridement and irrigation performed in the operating room (Fig. 1). Of patients treated with operative care, 12 (15.8%) patients received internal fixation for an associated articular fracture.

The mean age of the overall cohort was 35.2 ± 10.0 years, with no significant difference between the operative (36.1 ± 11.4 years) and nonoperative (34.5 ± 8.8 years) groups (*p* = 0.519). Most patients were male (85.7%) and of Hispanic ethnicity (72.7%), with similar distributions across both treatment groups. Other baseline characteristics, including body mass index (BMI), area deprivation index (ADI), and comorbid medical conditions, were also comparable between the groups, with no statistically significant differences observed in the treatments that patients received (Table 1).

**Table 1:** Demographic and comorbidity characteristics of patients with ballistic traumatic arthrotomy by treatment

	Overall *n* = 77	Nonoperative care *n* = 42	Operative care *n* = 35	*p*-value
Age (years)	35.2 ± 9.98	34.5 ± 8.75	36.1 ± 11.4	0.519
Sex				0.327
Female	11 (14.3%)	8 (18.2%)	3 (8.1%)	
Male	66 (85.7%)	34 (80.9%)	32 (91.4%)	
Race/Ethnicity				0.654
Hispanic	56 (72.7%)	30 (71.4%)	26 (74.3%)	
Black	8 (10.4%)	6 (14.2%)	2 (5.7%)	
Native American	1 (1.3%)	0 (0.0%)	1 (2.9%)	
Caucasian	5 (6.5%)	2 (4.8%)	3 (8.6%)	
Asian	1 (1.3%)	1 (2.4%)	0 (0.0%)	
Other	6 (7.8%)	3 (7.1%)	3 (8.6%)	
BMI (kg/m^2^)	28.7 ± 7.43	27.8 ± 7.29	29.8 ± 7.56	0.108
ADI	5.94 ± 1.71	5.82 ± 1.56	6.11 ± 1.91	0.500
Comorbid medical				
Conditions	33 (42.9%)	16 (38.1%)	17 (48.6%)	0.368
Tobacco use	3 (3.9%)	2 (4.8%)	1 (2.9%)	1.00
Diabetes	11 (14.3%)	5 (11.9%)	6 (17.1%)	0.534
Mental disorder	8 (10.4%)	6 (14.3%)	2 (5.7%)	0.280
Alcoholism	16 (20.8%)	9 (21.4%)	7 (20.0%)	1.00
Substance use	8 (10.4%)	2 (4.8%)	7 (20.0%)	0.131
Hypertension	21 (27.3%)	12 (28.6%)	9 (25.7%)	0.803
Other				
Payor				
Government	59 (76.6%)	32 (76.1%)	27 (77.1%)	1.00
Private	7 (9.1%)	4 (9.5%)	3 (8.6%)	
Other	11 (14.3%)	6 (14.3%)	5 (14.3%)	

^*^BMI, body mass index; Kg/m^2^, kilograms per meter^2^; HTN, hypertension; ADI: area deprivation index (state)

### Injury and treatment characteristics

The knee was the most frequently involved joint, accounting for 40.3% of the total cases, 51.4% of the operative case, and 31.0% of the nonoperative cases (Table 2). The elbow was the second most affected joint, representing 24.7% of cases, with similar distributions between treatment groups. No significant difference between the joint affected and the treatment was observed (*p* = 0.090). Cefazolin monotherapy was the most frequently method of antibiotic prophylaxis (Table 3).

**Table 2:** Joint involved with ballistic traumatic arthrotomy by treatment

	Overall *n* = 77	Nonoperative care *n* = 42	Operative care *n* = 35	*p*-value
Ankle	7 (9.1%)	5 (11.9%)	2 (5.7%)	0.090
Elbow	19 (24.7%)	10 (23.8%)	9 (25.7%)	
Hip	7 (9.1%)	4 (9.5%)	3 (8.6%)	
Knee	31 (40.3%)	13 (31.0%)	18 (51.4%)	
Shoulder	7 (9.1%)	7 (16.7%)		
Shoulder/Elbow	1 (1.3%)		1 (2.9%)	
Wrist	5 (6.5%)	3 (7.1%)	2 (5.7%)

**Table 3:** Antibiotic therapy for ballistic traumatic arthrotomy by treatment

	Overall *n* = 77	Nonoperative care *n* = 42	Operative care *n* = 35	*p*-value
Cefazolin	60 (77.9%)	31 (73.8%)	29 (82.9%)	0.563
Cefazolin+	7 (9.1%)	4 (9.5%)	3 (8.6%)	
Other other	10 (13.0%)	7 (16.7%)	3 (8.6%)	

### Outcomes

Overall infection occurred in eight patients (10.4%). Of those patients, four sustained fractures of the olecranon process, with two of those four with concurrent fracture of the proximal ulna. Two patients sustained tibial plateau fractures, of which one had extension of injury to the patellar tendon. The last two patients had a fracture to the glenoid fossa and a fracture of the distal humerus, respectively. The patient with a glenoid fossa fracture was treated with clindamycin for a three-day course and was found to have blood cultures positive for *Streptococcus pneumoniae*. One patient with an olecranon fracture was treated with a two day course of vancomycin at the surgeon’s discretion, and the remaining patients that developed infection were treated with cefazolin ranging from four days to fourteen days. Four of these patients reported tobacco use, two reported prior methamphetamine use confirmed by a urine toxicology report, two had a diagnosed mental health condition, and one had hypertension not controlled with medication. No other comorbidity was reported.

No significant difference was observed in the incidence of overall infection by treatment (7.1% with antibiotic therapy alone vs. 14.3% with adjunctive surgical debridement, *p* = 0.457; Table 4). BWI without involvement of the joint capsule was reported in 3 patients (3.9%), with a slightly higher occurrence in the operative group (5.7%) compared to the nonoperative group (2.4%, *p* = 0.830). SA occurred in five patients (6.5%). No significant difference was observed in the incidence of SA by treatment (4.8% vs. 8.5%, *p* = 0.995). Among the 12 patients who received operative care, 3 treated with fixation developed an infection versus 2 (25.0% vs. 8.7%, *p* = 0.594).

**Table 4:** Infectious complications of ballistic traumatic arthrotomy by treatment

	Overall *n* = 77	Nonoperative care *n* = 42	Operative care *n* = 35	*p*-value
Overall infection	8 (10.4%)	3 (7.1%)	5 (14.3%)	0.457
Ballistic wound infection	3 (3.9%)	1 (2.4%)	2 (5.7%)	0.830
Septic arthritis	5 (6.5%)	2 (4.8%)	3 (8.5%)	0.995

### Post-hoc power analysis

The power of this sample to detect a difference in the rate of the infectious outcomes was analyzed post-hoc. Forty-two patients in the nonoperative group and thirty-five in the operative group, with an incidence of 7.1% and 14.3% respectively provided a power of 15.9% for detecting a difference in infection between the two groups. Three hundred forty-one patients would be required in each group to detect a difference of 7.2% with a power of 80% and an alpha of 0.017 after correction for multiple comparisons with Šidák-Holm.

## Discussion

In this observational study at one urban Level 1 Trauma center, operative debridement of low velocity ballistic traumatic arthrotomy of large and intermediate joints as an adjunct to antibiotic therapy was not associated with ballistic wound infection nor septic arthritis compared to antibiotic therapy alone. The knee was the most frequently affected joint (40%, similar to prior studies of ballistic arthrotomies [7, 13], while the ankle was the site of ballistic traumatic arthrotomy most frequently treated with antibiotics alone (71.4%).

Recent studies have also questioned the necessity of routine surgical debridement for traumatic arthrotomy in general [20]. McKnight et al. performed a prospective observational study of 189 patients with traumatic arthrotomy of large and intermediate joints, 64 of which were treated nonoperatively with antibiotics and bedside closure and 125 were treated operatively [20]. Overall, 1.7% of the nonoperative group developed septic arthritis compared to 5.8% in the operative group (*p* = 0.270). No difference in the incidence of septic arthritis with operative care was observed when stratified for size of the traumatic arthrotomy. These results support the use of nonoperative treatment for small (< 50 mm), minimally contaminated traumatic arthrotomies. (< 50 mm), minimally contaminated traumatic arthrotomies.

Low-velocity ballistic arthrotomies may also not need formal debridement and irrigation to prevent infection. Shultz et al. performed a retrospective cohort review of 45 patients with a low velocity ballistic arthrotomy confirmed with a saline load test or gas within the joint affected on computed tomography scan [7]. Eight were treated with prophylactic antibiotics and bedside irrigation and 37 received formal debridement and irrigation. Neither group had an incidence of septic arthritis or deep infection. Each group had one case of superficial infection with an overall infection rate of 4.3%. This study, similar to ours, was underpowered to detect a difference of 9.9% [7]. Liu et al. performed a retrospective cohort review of 195 patients with low velocity ballistic arthrotomies [13]. Eighty patients were treated nonoperatively, 16 underwent formal debridement and irrigation in the operating room, and 99 received a formal debridement and irrigation with open reduction and internal fixation (ORIF) procedure. All 195 patients received local wound care and antibiotic therapy at the discretion of the consulting physician. No incidences of septic arthritis were seen and only six total patients had a documented infection, all in the debridement and irrigation plus ORIF group, for an overall infection rate of 3.1% [13]. Differences in the locations of the fractures between groups likely impacted the incidence of infection within each group. These findings and the present provide a modest base of support for the nonoperative treatment of ballistic arthrotomies in the absence of other surgical indications.

A confounding consideration is that some patients received a formal debridement and irrigation as adjunctive rather than primary treatment for arthrotomy because the associated fracture was indicated for ORIF. Surgical fracture fixation requires additional time and exposure while introducing implant material upon which biofilms may form, independently and causally increasing the risk of infection. [21, 22] Increasing severity of fracture and disruption of the joint capsule with periarticular open injuries are associated with higher rates of both deep infection and septic arthritis [23]. Liu et al. observed a significantly higher rate of infection (6%) in patients who received debridement, irrigation, and ORIF than patients treated nonoperatively (0%) for ballistic arthrotomies. As in the present study, the association of surgical care with greater but statistically insignificant differences in the incidence of infection may be confounded by greater severity of the fractures in the operative group that required internal fixation [13].

Nonoperative management of ballistic traumatic arthrotomy has substantial implications for patient experience, provider workload, and health system resource utilization. Obviating surgery of marginal incremental benefit may reduce patient morbidity, shorten hospital stays, and decrease healthcare costs [9, 20]. McKnight et al. as above found a statistically significant median cost saving of $10,8884 per patient treated nonoperatively, with the operative group having a median total cost of $11,973 [20]. Nonoperative care also reduces the risk of anesthetic and perioperative complications and the attendant drain on healthcare resources, which may be particularly relevant in settings with limited surgical capacity or during times of increased demand such as the COVID-19 pandemic [19]. High-volume urban trauma centers which manage the greatest volumes of ballistic trauma are likely to experience cost savings from the option of nonoperative care for traumatic arthrotomy, freeing operating room availability and resources for allocation to other patient care and operational needs.

The retrospective design inherently limits the ability to establish causality and may be subject to bias including outcome censorship, documentation inaccuracies, and missing data. The generalizability of these findings to other populations or healthcare settings may be limited as the relatively short follow-up period may not capture late-onset infections or long-term functional outcomes. A post-hoc power analysis confirmed that the study is underpowered to clearly prove a significant difference in infection rate with nonoperative treatment. Our study was powered to see a difference of 10%, but a difference of 7.2% in overall infection was observed between the two groups.

## Conclusion

Formal surgical debridement of ballistic traumatic arthrotomy of large and intermediate joints did not provide significant reductions in the rates of wound infection or septic arthritis management of ballistic traumatic arthrotomy in major joints resulting from low-velocity GSWs compared with antibiotic therapy alone. Evidence from this study supports the notion that nonoperative management does not increase the risk of septic arthritis, and management without formal debridement and irrigation may be sufficient. An appropriately powered randomized controlled trial of antibiotic therapy alone versus surgical debridement and antibiotics for patients with traumatic arthrotomy may help address this clinical question.

## Data Availability

No datasets were generated or analysed during the current study.
